# Complement receptor 1 (CR1, CD35) association with susceptibility to leprosy

**DOI:** 10.1371/journal.pntd.0006705

**Published:** 2018-08-09

**Authors:** Gabriela Canalli Kretzschmar, Luana Caroline Oliveira, Renato Mitsunori Nisihara, Thirumalaisamy P. Velavan, Sérvio Túlio Stinghen, Ewalda R. S. Stahlke, Maria Luiza Petzl-Erler, Iara José T. de Messias-Reason, Angelica Beate Winter Boldt

**Affiliations:** 1 Laboratory of Human Molecular Genetics, Department of Genetics, Federal University of Paraná, Curitiba, Brazil; 2 Laboratory of Molecular Immunopathology, Department of Clinical Pathology, Clinical Hospital, Federal University of Paraná, Curitiba, Brazil; 3 Institute of Tropical Medicine, Department of Human Parasitology, University of Tübingen, Tübingen, Germany; 4 Vietnamese- German Center for Medical Research, VG-CARE, Hanoi, Vietnam; 5 State Department of Health of Paraná, Curitiba, Brazil; University of Oklahoma, UNITED STATES

## Abstract

**Background:**

Pathophysiological mechanisms are still incompletely understood for leprosy, an urgent public health issue in Brazil. Complement receptor 1 (CR1) binds complement fragments C3b/C4b deposited on mycobacteria, mediating its entrance in macrophages. We investigated *CR1* polymorphisms, gene expression and soluble CR1 levels in a case-control study with Brazilian leprosy patients, aiming to understand the role of this receptor in differential susceptibility to the disease.

**Methodology:**

Nine polymorphisms were haplotyped by multiplex PCR-SSP in 213 leprosy patients (47% multibacillary) and 297 controls. mRNA levels were measured by qPCR and sCR1 by ELISA, in up to 80 samples.

**Principal findings:**

Individuals with the most common recombinant haplotype harboring rs3849266*T in intron 21 and rs3737002*T in exon 26 (encoding p.1408Met of the York Yk^a^+ antigen), presented twice higher susceptibility to leprosy (OR = 2.43, p = 0.017). Paucibacillary patients with these variants presented lower sCR1 levels, thus reducing the anti-inflammatory response (p = 0.040 and p = 0.046, respectively). Furthermore, the most ancient haplotype increased susceptibility to the multibacillary clinical form (OR = 3.04, p = 0.01) and presented the intronic rs12034383*G allele, which was associated with higher gene expression (p = 0.043), probably increasing internalization of the parasite. Furthermore, there was an inverse correlation between the levels of sCR1 and mannose-binding lectin (initiator molecule of the lectin pathway of complement, recognized by CR1) (R = -0.52, p = 0.007).

**Conclusions:**

The results lead us to suggest a regulatory role for *CR1* polymorphisms on mRNA and sCR1 levels, with haplotype-specific effects increasing susceptibility to leprosy, probably by enhancing parasite phagocytosis and inflammation.

## Author summary

The reasons for which some individuals resist *Mycobacteria leprae* infection, whereas others contract leprosy and only a subgroup of them become severely affected, are still poorly understood. The complement receptor 1 (CR1) serves as a gate for bacterial entry in macrophages, but its importance in the spread of infection and emergence of symptoms is unknown. Despite having many common structural and regulatory variants, the *CR1* gene was investigated only once in a leprosy association study in Malawi. In order to fill in this gap, we investigated if *CR1* polymorphisms are co-responsible for differential disease susceptibility in 213 leprosy patients and 297 controls, also measuring mRNA and soluble CR1 levels. Associations were dependent on specific combinations of variants in regulatory and coding regions, which were also associated with gene and protein expression. Thus, this study corroborates the importance of the CR1 receptor in the susceptibility to leprosy and is the first to bring information about *CR1* polymorphisms in the Brazilian population, as well as to show the relationship between genotypes and mRNA and sCR1 levels.

## Introduction

Leprosy has been reported for millennia in many ancient cultures, being currently common and causing great social stigma for affected individuals in India, Brazil, Indonesia, Bangladesh, Democratic Republic of Congo, Ethiopia, Nepal and Nigeria [1–3]. India, Brazil and Indonesia report more than 10 000 new leprosy patients, annually. Together, they figure up 81% of the newly diagnosed and reported patients, globally [4]. In Brazil, the state with the highest prevalence is Mato Grosso (9.03 cases / 10.000 inhabitants) [5].

Leprosy is a chronic infectious disease caused by *Mycobacterium leprae* and *M*. *lepromatosis* [6] that primarily affects the skin and peripheral nervous system, later reaching other organs and systems [7,8]. After being exposed to the pathogen, most of the individuals present resistance to infection. Those who develop the disease can fit within a broad clinical spectrum with two antagonistic poles, from paucibacillary tuberculoid to multibacillary lepromatous disease [9]. This clinical diversity relies on the quality of the immune response, which itself results from genetic variants of the host and environmental factors [10]. Genome-Wide Association Studies (GWAS), all done with the Chinese population, identified polymorphisms in interleukin 23 receptor (*IL23R*), nucleotide-binding oligomerization domain containing 2 (*NOD2*) and human leukocyte antigens–antigen D related (*HLA-DR*) genes as associated with susceptibility to the disease [11–14]. In addition, polymorphisms of genes associated with Parkinson (Parkin—*PARK2*, parkin coregulated—*PACRG* and superoxide dismutase 2 -*SOD2*) and Alzheimer diseases (*SOD2*) modulate susceptibility to leprosy in independent populations [15,16].

The complement system includes 47 proteins and protein fragments that activate, coordinate and regulate a proinflammatory, proteolytic cascade of the immune response [17]. Altered expression and functional deficiency of complement components modulate susceptibility to diseases. Genetic variants that reduce complement activation or complement-mediated recognition of opsonized elements may enhance resistance against pathogens that depend on phagocytosis to initiate infection, as mycobacteria. They may also decrease inflammation at skin and nerve injuries [18,19]. Indeed, several polymorphisms of genes of the complement system are associated with leprosy: mannose-binding lectin (*MBL2*), ficolins (*FCN1*, *FCN2* and *FCN3*), the serine protease associated with them (*MASP2*—mannose-binding lectin serine protease 2) and complement receptor 1 (*CR1*, also known as CD35) [10,20–24].

The entrance of mycobacteria into macrophages is mediated by complement receptors such as CR1, which binds C3b/C4b complement fragments deposited on opsonized bacteria [25]. CR1 is mainly expressed by erythrocytes, phagocytes, B and T cells, in membrane-bound or soluble form [26]. It accelerates the decay of C3 and C5 convertases and serves as a cofactor in factor I–mediated cleavage of C3b/C4b into ligands for other complement receptors [27]. In addition, CR1 competes with MASPs for the same binding sites on collectins and ficolins, which initiate the lectin pathway of complement. These molecules possibly act as opsonins, leading to CR1-mediated internalization of pathogens into phagocytes [28]. In malaria, CR1 generates rosettes between *Plasmodium falciparum* infected and noninfected erythrocytes and mediates sialic acid–independent cell invasion [29,30]. Despite this multitude of functions, genetic association studies of infectious diseases with *CR1* single nucleotide polymorphisms (SNPs) are scarce [20,31], and only one investigated this gene in leprosy, reporting a protective association with the McCoy^b^ allele (rs17047660) in a rural region of Malawi [20]. In this work, we aim to fill in this gap by investigating *CR1* polymorphisms, mRNA expression levels and sCR1 serum levels in Brazilian leprosy patients.

## Material and methods

### Ethics statement

This transversal case-control study was approved by The Ethics Committee of the Federal University of Paraná (Clinical Hospital) (protocols 218.104 and 279.970). All study participants were informed about the research and signed a term of informed consent.

### Subjects and samples

A total of 213 leprosy patients were recruited from three reference centers: Dermatology Service at the Clinical University Hospital–(UFPR), Regional Center for Metropolitan Specialties (both in Curitiba–PR, South Brazil) and Reference Center for Leprosy and Tuberculosis (Sinop–MT, Central-West Brazil), within a period of 10 years (2005–2015). Data for both patients and controls was collected through interviews and medical records. Cases were ascertained by the responsible dermatologists of each hospital/reference center. Patients were classified as either paucibacillary (PB) or multibacillary (MB), according to clinical presentation and bacilloscopy results, following recommendations of the World Health Organization (WHO). Individuals with confirmed clinical diagnosis of leprosy and older than 18 years were included. Patients with skin conditions other than leprosy (either autoimmune or infectious) were excluded. As controls, we included 297 healthy volunteers and blood donors from the same reference centers or nearby blood banks, that may share patient’s environmental factors, including exposure to the parasite. They were from Paraná Hematology Center–HEMEPAR (South Brazil), Reference Center for Leprosy and Tuberculosis (Sinop–MT) and Adventist Hospital of Pemphigus (Campo Grande–MS) (both in Central-Western Brazil). Individuals older than 18 years and without any pathological skin condition were included. In addition, all participants were classified according to ethnic origin, based on physical characteristics and self-reported ancestry, into Euro and Afro-Brazilian. This strategy has been confirmed by HLA genotyping, where an average sub-Saharan African component of 9% and an average Amerindian component of 5% was identified for the first, and at least 40% of African and 6% of Amerindian ancestry for the last [32,33]. Demographic characteristics of the participants of this study are listed in Table 1.

**Table 1:** Demographic data of leprosy patients and controls.

	Controls n = 326	Patients n = 213	Multibacillary n = 100	Paucibacillary n = 91
**South (%)**	225 (69.0)	144 (67.6)	86 (86)	37 (40.7)
**Central-Western (%)**	101 (31.0)	69 (32.4)	14 (14)	54 (59.3)
**Male (%)**	152 (46.6)	127 (59.6)	67 (67)	37 (40.7)
**Average age (min-max)**	40.02 (18–76)	49.1 (18–94)	51.5 (20–94)	46.7 (19–71)
**Euro-Brazilian (%)**	235 (72.1)	168 (78.9)	82 (82)	70 (76.9)
**Afro-Brazilian (%)**	91 (27.9)	45 (21.1)	18 (18)	21 (23.1)

n = number of individuals. South: Curitiba—PR; Central-Western: Sinop—MT and Campo Grande—MS. The total of multi- and paucibacillary patients did not sum 213, due to lack of proper diagnostic classification of 22 individuals.

### *CR1* genotyping

Blood was collected with and without anticoagulant ethylenediaminetetraacetic acid and DNA extracted from peripheral blood mononuclear cells through commercial kits (Qiagen, Hilden, Germany and GFX Genomic Blood DNA Purification Kit, GE Healthcare, São Paulo, Brazil). For samples from Campo Grande, MS, we used the phenol-chloroform-isoamyl alcohol protocol [34].

**Fig 1:**
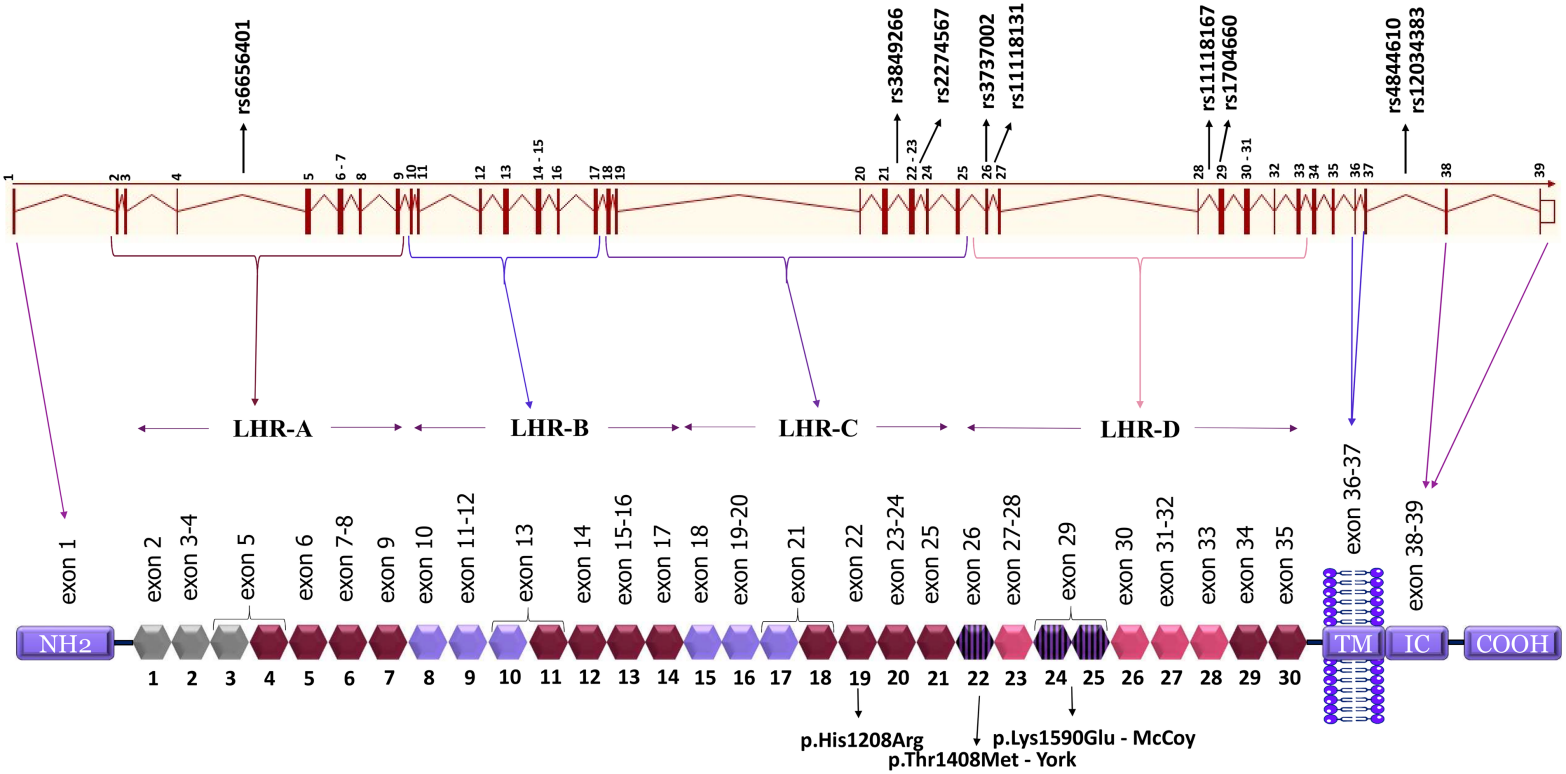
CR1 gene and protein structure and localization of the investigated SNPs. Each block in hexagon shape represents a SCR (short consensus repeat, numbered 1 to 30), encoded by the exons 2–35, shown just above. The other exons encode: the aminoterminal region (NH2, exon 1), transmembrane domain (TM, exons 36–37) and the intracytoplasmic carboxi-terminal domain (IC COOH, exons 38–39), which are all shown in blue. There are three binding sites for C4b opsonins (SCR 1–3, 8–10 and 15–1, in gray and lilac color), of which two are also C3b binding sites (SCR 8–10 and 15–17, both in lilac color). SCRs 22–28 bind C1q, MBL and ficolins (molecules that recognize either antibodies, sugar moieties or acetylated residues on the surface of the pathogen, respectively). SCRs 22, 24 and 25 (blue dashed blocks) carry York and McCoy blood group antigens frequent in Africans, but rare in Euro-descendants (downward pointing arrows indicate the responsible amino acid substitutions). The SNPs analysed in this study are indicated in the gene and the amino acid substitutions resulting from polymorphisms located in exons are indicated in the protein. The four long homologous repeats (LHR) of this protein are depicted: 1–7, 8–14, 15–21, 22–28. The functional sites of 8–10 and 15–17 are nearly identical. TM, transmembrane domain; IC, intracytoplasmic domain.

We genotyped nine SNPs: rs6656401 (*NC_000001*.*11*:*g*.*207518704A>G* in intron 4); rs3849266 (*NC_000001*.*11*:*g*.*207579645C>T* in intron 21); rs2274567 (*NC_000001*.*11*:*g*.*207580276A>G* in exon 22, exchanging histidine by arginine—NP_000564.2:p.His1208Arg); rs3737002 (*NC_000001*.*11*:*g*.*207587428C>T* in exon 26, exchanging threonine by methionine—NP_000564.2:p.Thr1408Met); rs11118131 (*NC_000001*.*11*:*g*.*207587851C>T* in intron 26); rs11118167 (*NC_000001*.*11*:*g*.*207608809T>C* in intron 28); rs17047660 (*NC_000001*.*11*:*g*.*207609511A>G* in exon 29, exchanging lysine by glutamic acid—NP_000564.2:p.Lys1590Glu); rs4844610 (*NC_000001*.*11*:*g*.*207629207A>C* in intron 37); rs12034383 (*NC_000001*.*11*:*g*.*207630250G>A* in intron 37) (Fig 1). They were selected by: association with any disease, being a tag SNP with r^2^ ≥ 0.8 in European (Utah–USA), Mexican, Colombian or Yoruba populations of the 1000 Genomes Project [35]; and/or with minor allele frequency (MAF) > 0.05. Noncoding SNPs were also chosen for investigation, due to their possible regulatory effect on gene expression.

We developed two multiplex Polymerase Chain Reaction—Single Specific Primer (PCR-SSP) reactions for simultaneous identification of four SNPs and a simple PCR-SSP for an isolated SNP. Each reaction coamplified a non-specific monomorphic fragment, for quality control. Primer sequences are shown in Table 2. All reactions were carried out in a final volume of 8 μl, containing 20 ng of genomic DNA, 0.2 Mm of each dNTP, 1x Coral Buffer (Qiagen, Hilden, DE). Thermal cycling began with 94°C for 5 min; followed by 33 (simple PCR-SSP and multiplex PCR-SSP 2) or 35 (multiplex PCR-SSP 1) cycles, where each cycle began with 94°C for 20s and ended with 72°C for 40s. We evaluated the amplified fragments after electrophoretic run on 1% agarose gel, stained with Sybrsafe (Invitrogen Life Technologies, Carlsbad, CA) (S1 Fig).

**Table 2:** Sequence-specific primers* for selected *CR1* polymorphisms.

FORWARD PRIMERS				REVERSE PRIMERS			
	SNP	Region	5’→3’ Sequence	SNP	Region	5’→3’ Sequence	bp
**Simple PCR**	rs6656401	Intron 4	CTCTGTCTCCATCTTCTC**A**	Generic Primer	Intron 4	CATAGTTGTAGTTGGGGATTG	257
			CTCTGTCTCCATCTTCTC**G**				
**Multi.2**	rs3849266	Intron 21	CTGATGGCTTGGGGTA**T**	rs2274567	Exon 22	CTCAATCTGCATTGATCCA**C**	667
			CTGATGGCTTGGGGTA**C**			CTCAATCTGCATTGATCCA**T**	
**Multi.2**	rs4844610	Intron 37	CTACACAAAACAGCCTTGT**A**	rs12034383	Intron 37	AGATGTCCATGCCTTAA**C**	1080
			CTACACAAAACAGCCTTGT**C**			AGATGTCCATGCCTTAA**T**	
**Multi.1**	rs3737002	Exon 26	CCATTTGCCAGTCCTA**C**	rs11118131	Intron 26	CAAGAAGAAGGGGTGAT**G**	457
			CCATTTGCCAGTCCTA**T**			CAAGAAGAAGGGGTGAT**A**	
**Multi.1**	rs11118167	Intron 28	GCCAATATGTGAATATTATTATCTTA**T**	rs17047660	Exon 29	TTCTGGAGCTGTGCATT**T**	746
			GCCAATATGTGAATATTATTATCTTA**C**			TTCTGGAGCTGTGCATT**C**	
**Control Primers**							
**Multi. 2 / Simple PCR**		HGH f	TGCCTTCCCAACCATTCCCTTA		HGH r	CCACTCACGGATTTCTGTTGTGTTTC	431
**Multi. 1**		HLA-E f	CGGGACTGACTAAGGGGCGG		HLA-E r	GTAGCCCTGTGGACCCTCTTAC	324

* excepting the generic and control primers. In bold: variant nucleotide.

Multi: multiplex; SNP: single nucleotide polymorphism; bp: base pairs; f: forward; r: reverse; HGH: Human Growth Hormone; HLA-E: Human Leukocyte Antigen E

For the simple PCR-SSP, where we discriminated the rs6656401 alleles, annealing temperatures were 58°C for the initial 11 cycles, 55°C for the following 11 cycles and 52°C for the last 11 cycles. We used 0.3 μM of each SSP and 0.1 μM of each control primer, 1.5 mM MgCl_2_ and 0.04 units of Taq polymerase (Invitrogen Life Technologies, Carlsbad, CA).

In the PCR-SSP multiplex 1, annealing temperatures were 59°C for the initial 8 cycles, 57°C for the following 7 cycles, 55°C for another 10 cycles and 53°C for the last 10 cycles. We used 0.5 μM of SSPs for rs3737002 and rs11118131, 0.6 μM for rs11118167 and rs17047660 and 0.08 μM of each control primer, 1.5 mM MgCl_2_ and 0.2 units of Platinum Taq DNA polymerase (Invitrogen Life Technologies, Carlsbad, CA).

In the PCR-SSP multiplex 2, annealing temperatures were 63 °C for the initial 6 cycles, 61°C for the following 16 cycles, 59°C for the last 11 cycles. We used 0.18 μM of SSPs for rs3849266 and rs2274567, 0.4 μM for rs4844610 and rs12034383, 0.08 μM of each control primer, 2 mM MgCl_2_ and 0.3 units of Taq polymerase (Invitrogen Life Technologies, Carlsbad, CA).

All protocols are available in protocols.io in the following dx.doi.org/10.17504/protocols.io.p49dqz6; dx.doi.org/10.17504/protocols.io.p5sdq6e; dx.doi.org/10.17504/protocols.io.p44dqyw.

### mRNA quantification

We collected blood from 33 controls and 46 leprosy patients (87% of which presented with the paucibacillary clinical form), all from Sinop, using tubes of the PAXgene Blood RNA system and extracted total stabilized RNA with the PAXgene Blood RNA Kit (both from PreAnalytiX, QIAGEN / BD). The RNA was reverse transcribed into cDNA with the High Capacity cDNA Reverse Transcription Kit (Applied Biosystems). mRNA levels were measured by quantitative real-time TaqMan PCR in ViiA 7 Real-Time PCR System (Applied Biosystems) and the TaqMan inventoried assay Hs00559348_m1, with a probe specific for the exon-exon junction encoding the CR1 domain most proximal to the cell membrane, common to the most abundant *CR1* mRNA transcripts. All assays were conducted in duplicate and the relative mRNA levels were normalized to mRNA expression of the beta-glucuronidase gene (*GUSB*) using the TaqMan inventoried assay 4333767F. Cq values (threshold cycle) were calculated using the ViiA 7 Software v1.2 (Applied Biosystems, USA), and gene expression with the comparative Cq method 2-ΔΔCq [36].

### sCR1 quantification

We measured sCR1 levels in serum of 58 leprosy patients (30 multibacillary and 28 paucibacillary) and 22 healthy controls from both Curitiba and Sinop, with similar ethnic distribution and presenting alleles associated with the disease. sCR1 levels were quantified by ELISA using SEB123Hu kit (USCN Life Science Inc., Wuhan, China), according to the manufacturer’s instructions. The sCR1 levels were correlated with previously measured MBL levels in 30 of the same patients, all from Curitiba [21].

### Statistical analysis

Allele, genotype and haplotype frequencies were obtained by direct counting. We calculated the sample size needed for detecting associations with allele/haplotype frequencies of at least 10% with 95% confidence level and a confidence interval of 5.0, arriving at minimal 384 chromosomes (at least 192 individuals). Thus, our work has enough statistical power for detecting an association with common alleles/haplotypes and susceptibility to leprosy *per se*, although not necessarily with clinical leprosy forms, since multibacillary and paucibacillary leprosy groups encompassed less individuals (100 and 91, respectively). Distribution of polymorphisms and haplotypes between patients and controls, multibacillary and paucibacillary patients, were compared using exact Fisher test (for haplotype frequencies) and binary multivariate logistic regression (for the dominant and recessive models) with STATA v.9.2 (Statacorps, Lakeway Drive, TX), correcting if necessary, for age, sex, geographic origin and ethnic group distribution. To correct for false discovery rate (q value), we used the Benjamini and Hochberg [37] approach on all significant results. The hypothesis of Hardy-Weinberg equilibrium was evaluated with the exact test of Guo & Thompson, implemented in Arlequin v.5.1. Linkage disequilibrium was evaluated with Haploview 4.2 [38]. Extended haplotypes were manually reconstructed based on linkage disequilibrium and phase information (obtained by the PCR-SSP amplification). Quantitative variables (CR1 mRNA and soluble CR1 levels) were not normally distributed and thus compared using nonparametric methods. They were grouped into patients and controls, multi- and paucibacillary patients, to check if levels alter according to disease status and severity. They were also grouped according to genotypes, to see if polymorphisms influence gene and protein expression. Data were transformed into log10 for better graphical visualization. Normality tests (D’Agostino & Pearson and Shapiro-Wilk test), correlation tests (Spearman) and non-parametric comparisons between medians of *CR1* mRNA and sCR1 levels (with Mann-Whitney and Kruskal-Wallis tests) were done using GraphPad Prism v.6 Software (GraphPad Software, La Jolla, CA).

## Results

### *CR1* polymorphisms and susceptibility to leprosy

**Fig 2:**
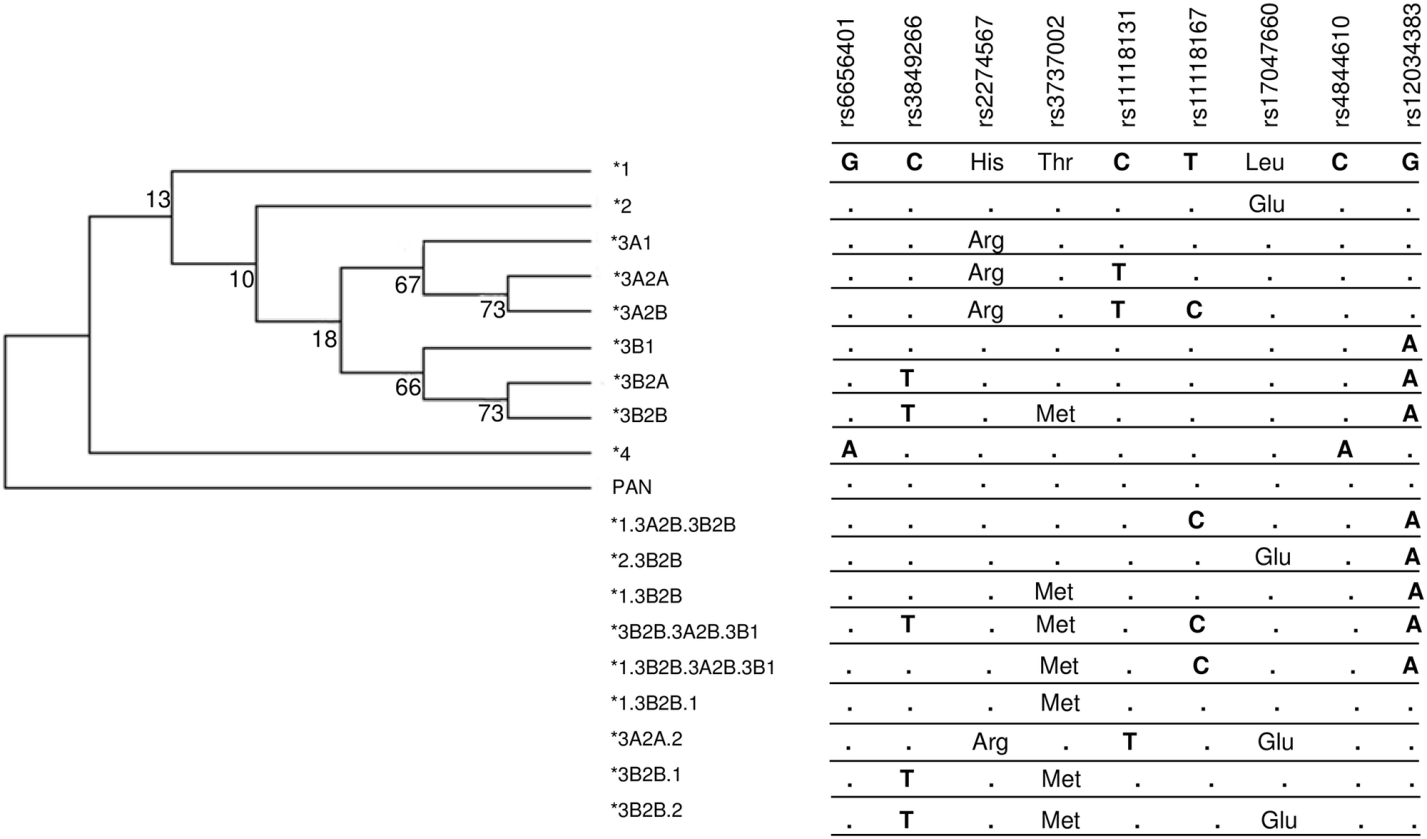
Maximum parsimony tree of *CR1* haplotypes with nucleotide changes and phylogenetic nomenclature.

Genotype distribution of controls followed the predictions of Hardy-Weinberg equilibrium. We were able to reconstruct haplotypes based on phase information obtained with SSP amplification and found 18 haplotypes, half of which possibly are recombinants. We confirmed the haplotypes by evaluating linkage disequilibrium (LD) in Euro-and Afro-Brazilians and named them according to the phylogenetic nomenclature suggested by others [39], adapted for recombinant haplotypes [40] (Fig 2, S2 Fig). The most ancestral *1 haplotype is identical to the sequence found in *Pan troglodytes* (NM_001193675). Haplotype *2 and three recombinant haplotypes (*2.*3B2B*, *3A2A.*2* and *3B2B.*2*) are unique in that they harbor the *G* allele in exon 29, encoding p.1590Glu, responsible for the McCoy McC^b^ blood group antigen (rs17047660). On the other hand, all haplotypes of clade *3A and one recombinant (*3A2A.*2*) encode p.1208Arg, due to the *G* allele in exon 22 (rs2274567). Yet clade *3B harbors all haplotypes with the *A* allele in intron 37 (rs12034383). They are probably involved in the generation of most recombinant haplotypes, reaching together a frequency higher than 40% (Table 3). For example, the York Yk^a^+ blood group antigen (corresponding to the *T* allele in exon 26 or p.1408Met), is solely encoded by one haplotype of this clade (*3B2B), but also by six recombinant haplotypes (*1.*3B2B*, *1*.*3B2B*.*1*, *1.*3B2B*.*3A2B*.*3B1*, *3B2B.*1*, *3B2B.*2* and *3B2B.*3A2B*.*3B1*). The *4 haplotype seems to be a relatively recent *in cis* combination, occurring on an isolated branch and harboring Alzheimer’s disease risk alleles rs6656401*A and rs4844610*A [41].

**Table 3:** Frequencies of *CR1* haplotypes.

Phylogenetic Haplotypes	Nucleotide sequence	Patients		Controls		OR	95% CI	P (q)	MB		PB		OR	95% CI	P (q)
		n	%	n	%				n	%	n	%			
*1	**GCHTCTLCG**	40	9.4	60	10.1			n.s.	26	13.0	10	5.5	3.04	1.31–7.07	0.01 (0.025)
*2	GCHTCTECG	12	2.8	14	2.4			n.s.	7	3.5	4	2.2			n.s.
*3A1	GCRTCTLCG	12	2.8	12	2.0			n.s.	4	2.0	6	3.3			n.s.
*3A2A	GCRTTTLCG	41	9.6	63	10.6			n.s.	21	10.5	17	9.3			n.s.
*3A2B	GCRTTCLCG	30	7.0	53	8.9			n.s.	14	7.0	12	6.6			n.s.
*3B1	GCHTCTLCA	75	17.6	118	19.9			n.s.	32	16.0	36	19.8			n.s.
*3B2A	GTHTCTLCA	17	4.0	17	2.9			n.s.	9	4.5	8	4.4			n.s.
*3B2B	GTHMCTLCA	69	16.2	111	18.7			n.s.	26	13.0	34	18.7			n.s.
*4	ACHTCTLAG	74	17.4	83	14.0			n.s.	35	17.5	31	17			n.s.
*1.*3A2B*.*3B2B*	GCHTCCLCA	7	1.6	11	1.9			n.s.	4	2.0	2	1.1			n.s.
*2.*3B2B*	GCHTCTECA	1	0.2	0	0			n.s.	0	0	1	0.5			n.s.
*1.*3B2B*	GCHMCTLCA	10	2.3	27	4.5			n.s.	5	2.5	4	2.2			n.s.
*3B2B.*3A2B*.*3B1*	**GTHMCCLCA**	22	5.2	14	2.4	2.43	1.18–5.03	0.017 (0.038)	11	5.5	8	4.4			n.s.
*1.*3B2B*.*3A2B*.*3B1*	GCHMCCLCA	1	0.2	0	0			n.s.	1	0.5	0	0			n.s.
*1.*3B2B*.*1*	GCHMCTLCG	3	0.7	6	1.0			n.s.	2	1.0	2	1.1			n.s.
*3A2A.*2*	GCRTTTECG	1	0.2	1	0.2			n.s.	0	0	1	0.5			n.s.
*3B2B.*1*	GTHMCTLCG	7	1.6	4	0.7			n.s.	3	1.5	2	1.1			n.s.
*3B2B.*2*	**GTHMCTECG**	4	0.9	0	0		n.a.	0.03 (0.05)*	0	0	4	2.2			n.s.
	Total	426	100	594	100				200	100	182	100			.

Haplotypes represented all nine investigated *CR1* SNPs, in the following order: rs6656401, rs3849266, rs2274567, rs3737002, rs11118131, rs11118167, rs17047660, rs4844610 and rs12034383. One-letter, underlined amino acid symbols are given instead of nucleotide substitutions, where appropriate. In bold: associated haplotypes. n: number of chromosomes; OR: odds ratio for the dominant model in binary logistic regression, corrected for age, ethnic group and gender distribution; CI: confidence interval; n.s. not significant; n.a.: not applicable.; MB: multibacillary, PB: paucibacillary; q: Benjamini-Hochberg corrected p-value;

*Fisher’s exact test.

The tree was rooted on the haplotype presenting high sequence identity with *Pan troglodytes* (NM_001193675). In the phylogenetic nomenclature system, the first clades to diverge are numbered with Arabic numerals. Sublineages of each clade are subsequently designated with capital letters and individual present-day haplotypes are given Arabic numerals, following the schema numerals/letters/numerals, if they diverge further. Recombinants are named according to the most common inferred parental haplotypes, separated by a dot, and citing the most similar parental haplotype, first. Bootstrap values are given on the respective branches. Amino acids are given where nucleotide substitution caused a missense mutation. Dendrogram was constructed following the maximum parsimony method using Mega Software v.6.

Among the recombinant haplotypes, the frequency of those sharing the initial *GTHMC* combination of the *3B2B haplotype—*3B2B.*3A2B*.*3B1*, *3B2B.*1* and *3B2B.*2* (where the last “HM” mean wild type p.1208Hist and the York Yk^a^+ p.1408Met amino acids), was higher in the patient group, than controls (33/426 or 7.7% vs. 18/594 or 3%, OR = 2.69 [95%CI = 1.49–4.84], Fisher’s exact test p = 0.0011, q = 0.0125). Individuals with the most common of them, the recombinant *3B2B.*3A2B*.*3B1* haplotype, presented twice higher susceptibility to leprosy *per se*, than controls, independent of age, gender and ethnicity (OR = 2.43, p = 0.017, q = 0.0375). Among leprosy patients, those with the *1 haplotype had an increased susceptibility to the more severe multibacillary clinical forms (OR = 3.04, p = 0.01, q = 0.025) (Table 3).

### *CR1* mRNA and sCR1 expression

**Fig 3:**
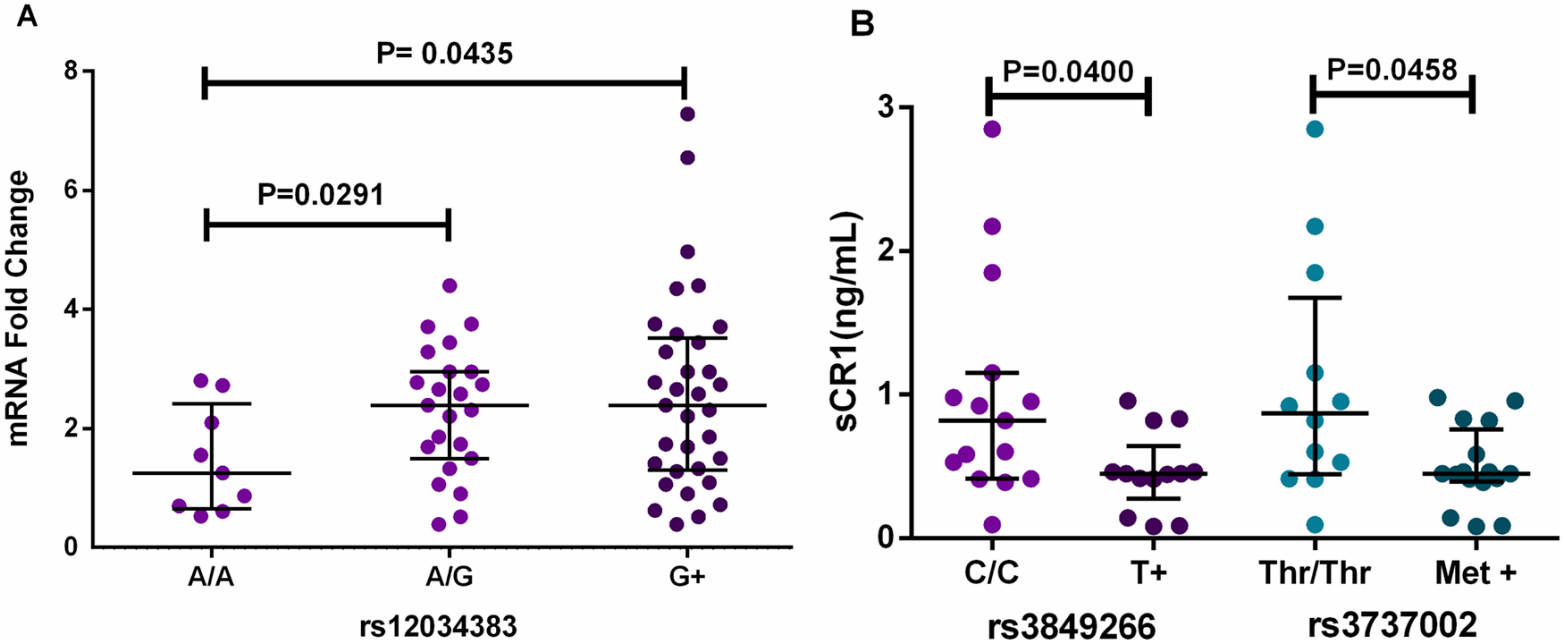
Association between *CR1* mRNA and sCR1 levels and CR1 polymorphisms. A Patients with the rs12034383*G allele presented higher mRNA expression, than *A/A* homozygotes. *G+*: *G* carriers. Excluded outliers: one (excluded for better visualization, inclusion did not change significance of the results). B: Paucibacillary patients have lower sCR1 concentration, if presenting the *T* allele of rs3849266 or Thr at rs3737002. *T+*: *T* carriers, Thr: threonine, Met: Methionine. Median, maximum and minimum values are in the Electronic Supplementary Material (S1 and S2 Tables). Scatter plots were constructed from the raw non-normalized, fold-change data. Comparisons were done with Mann-Whitney and Kruskal-Wallis tests, using GraphPad Software Prism v.6.

**Fig 4:**
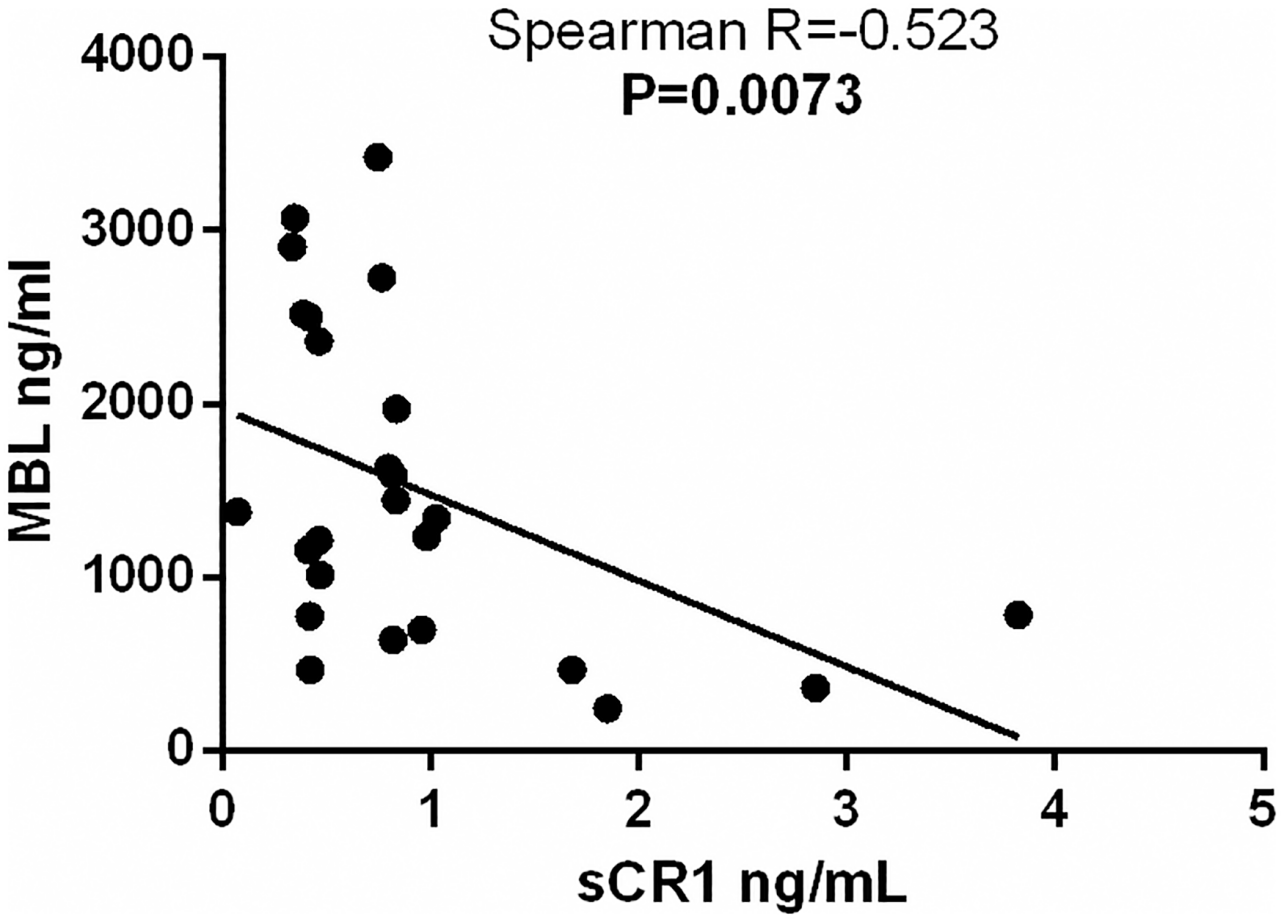
Negative correlation between MBL and sCR1 levels. MBL: mannose-binding lectin; sCR1: soluble complement receptor 1. Scatter plot was constructed from the raw non-normalized, fold-change data, using GraphPad Software Prism v.6.

As expected, we found no correlation between mRNA and sCR1 levels, since sCR1 is the result of enzymatic protein cleavage. Nevertheless, both were associated with intronic SNPs. Patients that carry the rs12034383*G allele presented higher mRNA expression than *A/A* homozygotes (p = 0.043) (Fig 3A). Furthermore, paucibacillary patients with the rs3849266 and rs3737002 *T* allele presented a reduction in sCR1 levels, compared with *C/C* (p = 0.040 and p = 0.046, respectively) (Fig 3B). Finally, there was a negative correlation between sCR1 and MBL serum concentrations in the patient group (Fig 4). Neither CR1 mRNA expression nor sCR1 concentration differed between patients and controls, or between clinical types (S1 and S2 Tables).

## Discussion

More than two decades ago, complement receptor CR1 was identified as a mediator for *Mycobacterium leprae’s* entry into phagocytes [25]. Despite the importance that *CR1* polymorphisms may have in the establishment of infection, only one association study on five missense mutations in this gene, has been conducted [20]. Although the genome-wide association study done with the Chinese population did not reveal any association of leprosy with *CR1*, it should be noted that this population has a markedly different genetic background from other populations, such as Brazilians [11–14]. Furthermore, as occurs with other genes encoding erythrocyte proteins, the *CR1* polymorphism has been modified by natural selection for malaria resistance in populations of endemic regions [29,41]. The present study is actually the first attempt to specifically analyse the influence of *CR1* haplotypes, mRNA expression and sCR1 levels in the susceptibility to leprosy.

**Fig 5:**
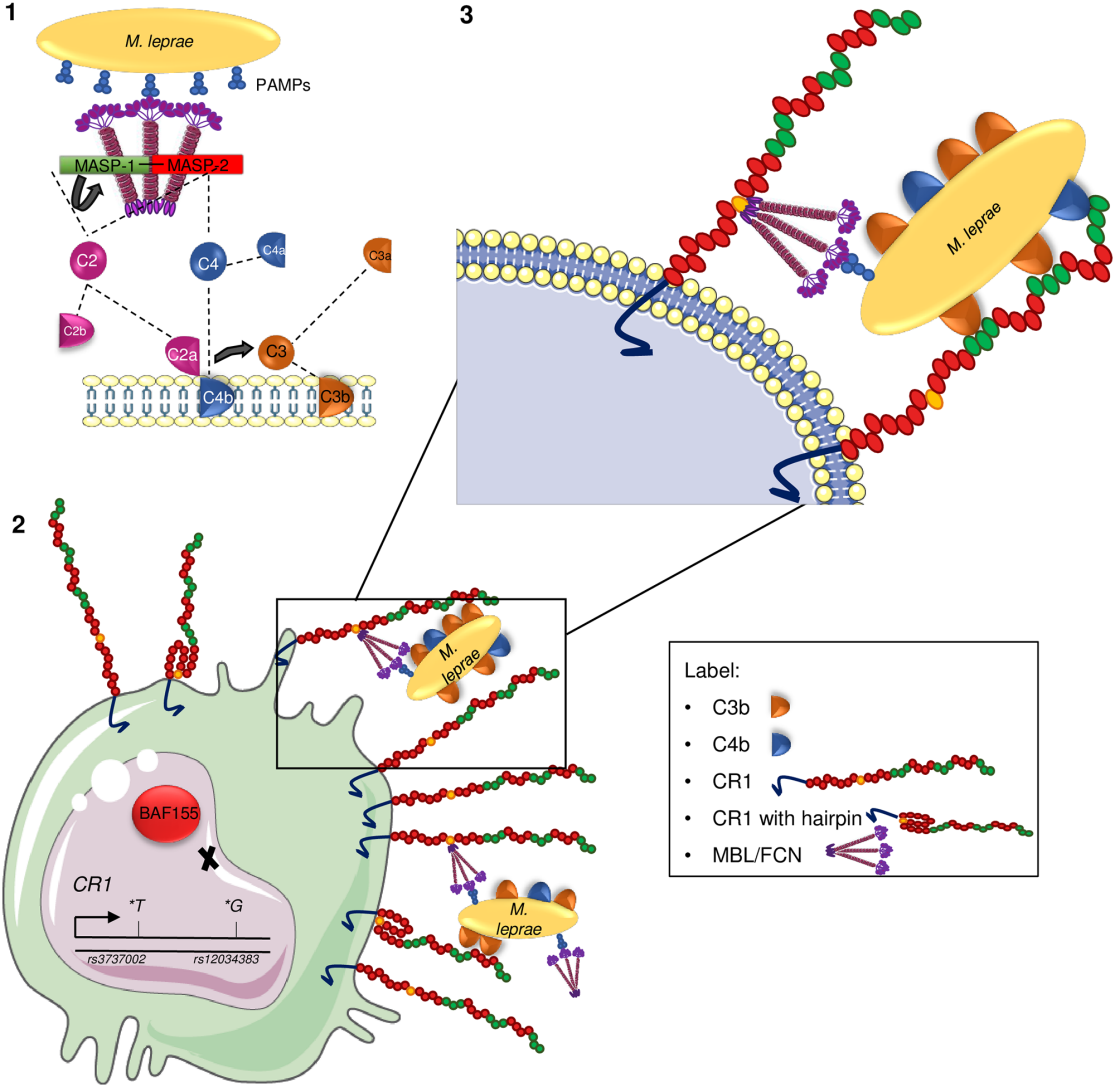
CR1 possible roles on leprosy susceptibility. (1) Pathogen or damage-associated molecular sugar patterns (PAMPs/DAMPs) on *Mycobacterium leprae* are recognized by mannose-binding lectin (MBL) or ficolins (FCNs) and activate the lectin pathway of complement. Autoactivation of the serine protease MASP-1 is followed by transactivation of MASP-2, cleavage of C2 and C4 and formation of the C3 convertase C2aC4b. C3 convertase ultimately leads to the insertion of C3b opsonins on the surface of the pathogen. (2) Macrophages presenting rs3737002*T (p.1408Met) may produce CR1 molecules whose enzymatic cleavage site is hidden, producing less soluble CR1 molecules. Those with rs12034383*G present lower affinity for BAF155-containing SWI/SNF nucleosome remodelling complex, increasing *CR1* gene expression. (3) Increased CR1 abundance is expected to enhance the uptake of C3b/C4b/ficolin/MBL-opsonized *M*. *leprae*, through the respective SCR (short consensus repeat) domains (shown in green and yellow).

We found an association of recombinant haplotypes sharing rs3849266*T and rs3737002*p.*1408Met* with two-fold higher susceptibility to leprosy. The rs3849266 SNP is located in a regulatory region bound by CTCF (CCCTC binding factor), as well as nine other regulatory proteins [42,43]. CTCF separates chromatin domains, regulating transcription [44]. Mutations in the DNA sequence recognized by this protein have been associated with cancer, most probably due to an interference with epigenetic regulation [44]. Yet rs3737002 is a missense variant (p.Thr1408Met) with a high Polyphen score of damage (1.00), configuring the York blood group antigen [45]. Paucibacillary patients with the rs3849266*T and p.1408Met alleles presented a reduction in sCR1 levels. It is possible that the York antigen alters protein conformation and hampers C-terminal cleavage of the CR1 transmembrane region [46] (Fig 5). The reason for which the leprosy susceptibility effect is restricted to recombinant haplotypes, not including the original *3B2B haplotype, leads us to suggest that the association relies on the combined effects of all other alleles configuring this haplotype, including hitch-hiking linked SNPs, not investigated in this study. We did also not find the protective association against leprosy, previously reported in a rural area of northern Malawi with the McCoy p.1590E blood group antigen [20]. Discrepant results are most probably due to ethnic differences, since our population was mainly composed of Euro-Brazilians (more than 70%).

The *1 haplotype, most similar to the ancestral *CR1* sequence, increased susceptibility to multibacillary disease. Multibacillary disease is known to be associated with a genetic susceptibility to build a Th2 immune response against mycobacterial spread, whereas susceptibility to leprosy *per se* is expected to be associated simply with genetic susceptibility to mycobacterial challenge (based especially on the quality of the innate immune response). Thus, it should not be surprising to find different *CR1* haplotypes associated with susceptibility to both conditions: CR1*1 with the first and CR1*3B2B.*3A2B*.*3B1*, with the second.

The *1 haplotype presents rs12034383*G in intron 37, which was associated with higher mRNA expression and is predicted to reduce the affinity for BAF155, a subunit of the SWI/SNF complex [42] (Fig 5). This complex regulates transcription by altering nucleosomal structure, using ATP hydrolysis [47]. Homozygotes for the rs12034383*A allele may present higher chromatin condensation than carriers of the *G* allele, reducing *CR1* gene expression. The *A* allele was indeed associated with lower *CR1* gene expression in different tissues of healthy individuals (https://www.gtexportal.org/home/snp/rs12034383). Even so, the expression results in this study were obtained with a majority of paucibacillary patients. They shall thus be cautiously interpreted, especially regarding multibacillary leprosy. The *A* allele occurs in the most frequent haplotypes, belonging to the phylogenetic clade *3B. It is associated with about 20% increased amyloid Aß cerebrospinal fluid levels in homozygote individuals, increasing susceptibility to Alzheimer’s disease [48]. It was also associated with higher erythrocyte sedimentation rate, a measure of ongoing inflammation [49].

Finally, higher sCR1 levels correlated negatively with MBL levels, previously measured [21]. Thus, it may be speculated that not only low MBL levels, but also increased levels of the inhibitory sCR1 protein, may be the products of an immune response regulation, that contribute to reduce inflammation and complement activation of the lectin pathway, at least among leprosy patients. This anti-inflammatory effect can be highly beneficial, protecting against lepromatous disease. Since sample size was very small, this hypothesis shall be further tested in other settings.

In conclusion, we suggest that *CR1* polymorphisms and haplotypes enhance susceptibility to leprosy by modulating gene expression and sCR1 abundance, to increase inflammation and parasite phagocytosis. Functional investigations on phagocytic efficiency of the associated haplotypes would help to clarify the role of this receptor in the disease and possibly lead to novel therapeutic strategies.

## Accession numbers/ID numbers for *CR1* gene and protein

NCBI: 1378, MIM: *120620, ENSEMBL: ENSG00000203710, hprd: 00398, UniProtKB: P17927.

## Supporting information

S1 Fig Simple PCR-SSP and multiplex PCR-SSP results of *CR1* SNPs. **A**- PCR-SSP for rs6656401 (*1*:*g*.*207518704A>G*) (specific fragment of 257 bp): 1) *G/G*, 2) *A/G*, 3) *A/G*, 4) *G/G*. **B**. PCR-SSP for rs3849266 (*1*:*g*.*207579645C>T*), rs2274567 (*1*:*g*.*207580276A>G*) (specific fragment of 667 bp) and rs4844610 (*1*:*g*.*207629207A>C*), rs12034383 (*1*:*g*.*207630250G>A*) (specific fragment of 1080 bp): 1) *CACA/TACA*; 2) *CGCG/TACA*; 3) *CAAG/CACG*; and 4) *CAAG/CACG*. **C**. PCR-SSP for rs3737002 (*1*:*g*.*207587428C>T*), rs11118131 (*1*:*g*.*207587851C>T*), (specific fragment of 457 bp); rs11118167 (*1*:*g*.*207608809T>C*), rs17047660 (*1*:*g*.*207609511A>G*): 1) (specific fragment of 746 bp): 1) *TCCA/TCTA*; 2) *CTCA/TCTA*; and 3) *CCCA/CTTA*. H: negative controls (complete reactions without DNA); bp: base pairs; HGH: Human Growth Hormone (as internal PCR control fragment of 431 bp for A and B); *HLA*-E: Human Leukocyte Antigen–E (as internal PCR control fragment of 324 bp).
(PDF)

S2 Fig Linkage disequilibrium between the investigated *CR1* polymorphisms. Colors are indicative of D'/logarithm of odds (LOD), and values correspond to r^2^. Bright red color represents LOD score for LD ≥ 2 and D = 1, shades of pink/red represents LOD ≥ 2 and D < 1, blue color represents D = 1 but LOD < 2, and white squares represent LOD< 2 and D < 1. A) Euro-Brazilians controls; B) Euro-Brazilians patients; C) Afro-Brazilian controls; D) Afro-Brazilian patients. Plots were constructed using Haploview 4.2.
(PDF)

S1 Table *CR1* gene expression in leprosy. Pat.: Patient; Con.: control; MB: multibacillary; PB: paucibacillary; N: samples; min.: minimum; med.:median; max.: maximum; P: P value for non parametric Mann-Whitney test; *G +* and *AA* correspond to SNP rs12034383. In bold: significant.
(PDF)

S2 Table Concentration of soluble CR1 in leprosy. Pat.: Patient; Con.: control; MB: multibacillary; PB: paucibacillary; N: samples; min.: minimum; med.:median; max.: maximum; P: P value for non parametric Mann-Whitney test; In bold: significant.
(PDF)

S3 Table Original *CR1* genotyping, mRNA and protein levels data used for logistic regression analysis. (XLSX)

S1 Checklist STROBE (Strengthening the Reporting of Observational studies in Epidemiology) checklist for a case-control study. (PDF)
