# Supplementary material for: Complement receptor 1 (CR1, CD35) association with susceptibility to leprosy
Source: PLoS Negl Trop Dis. 2018 Aug 9;12(8):e0006705. doi: 10.1371/journal.pntd.0006705 (PMC6103516; doi:10.1371/journal.pntd.0006705)
Supplement: S1 Fig — A- PCR-SSP for rs6656401 (1:g.207518704A>G) (specific fragment of 257 bp): 1) G/G, 2) A/G, 3) A/G, 4) G/G. B. PCR-SSP for rs3849266 (1:g.207579645C>T), rs2274567 (1:g.207580276A>G) (specific fragment of 667 bp) and rs4844610 (1:g.207629207A>C), rs12034383 (1:g.207630250G>A) (specific fragment of 1080 bp): 1) CACA/TACA; 2) CGCG/TACA; 3) CAAG/CACG; and 4) CAAG/CACG. C. PCR-SSP for rs3737002 (1:g.207587428C>T), rs11118131 (1:g.207587851C>T), (specific fragment of 457 bp); rs11118167 (1:g.207608809T>C), rs17047660 (1:g.207609511A>G): 1) (specific fragment of 746 bp): 1) TCCA/TCTA; 2) CTCA/TCTA; and 3) CCCA/CTTA. H: negative controls (complete reactions without DNA); bp: base pairs; HGH: Human Growth Hormone (as internal PCR control fragment of 431 bp for A and B); HLA-E: Human Leukocyte Antigen–E (as internal PCR control fragment of 324 bp). (PDF) [file pntd.0006705.s001.pdf]

ELETRONIC SUPPLEMENTARY MATERIAL

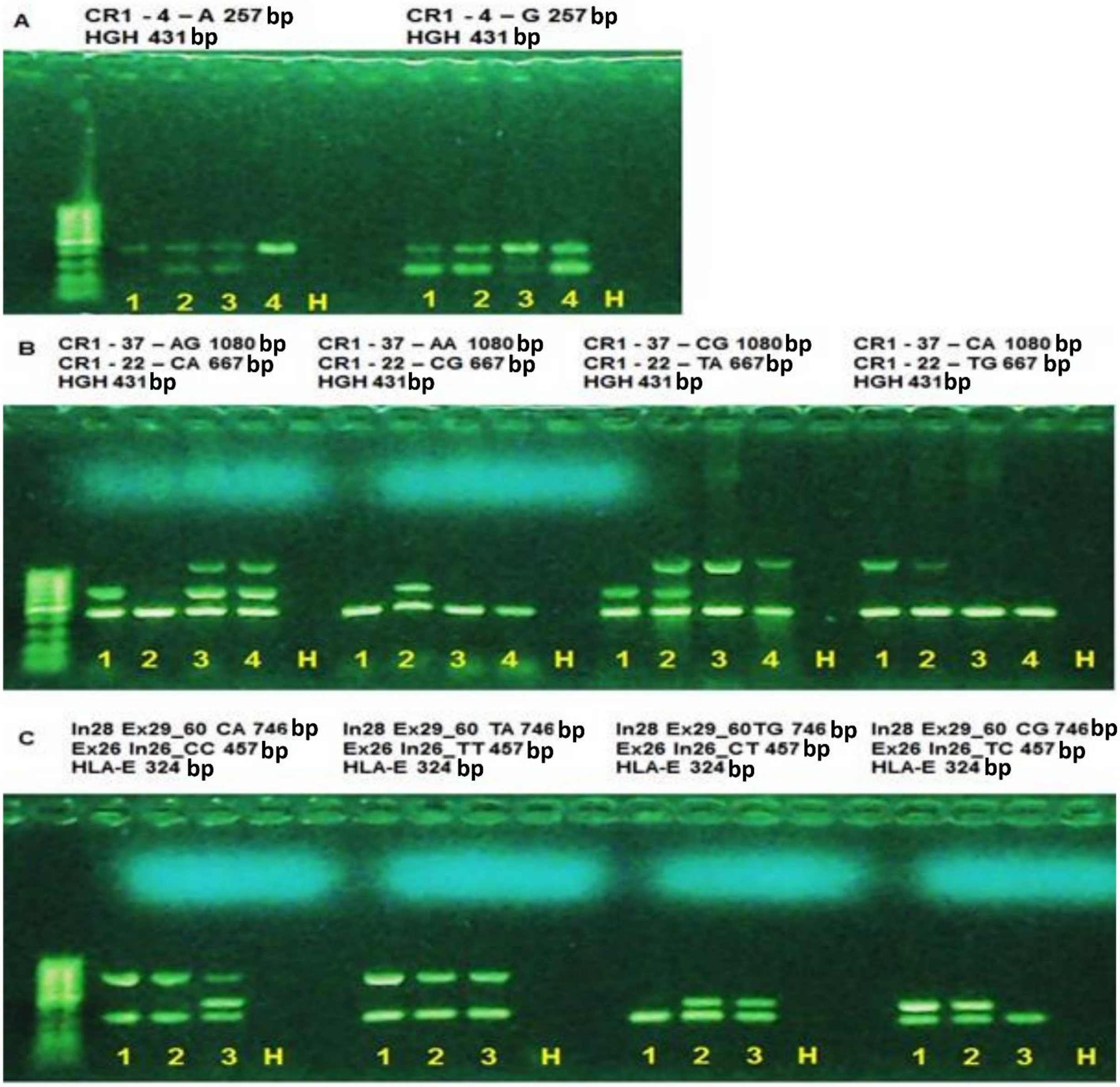

S1 Figure. Simple PCR-SSP and Multiplex PCR-SSP results of *CR1* SNPs.

A- PCR-SSP for rs6656401 (*1:g.207518704A>G*) (specific fragment of 257 bp): 1) *G/G*, 2) *A/G*, 3) *A/G*, 4) *G/G*. **B.** PCR-SSP for rs3849266 (*1:g.207579645C>T*), rs2274567 (*1:g.207580276A>G*) (specific fragment of 667 bp) and rs4844610 (*1:g.207629207A>C*), rs12034383 (*1:g.207630250G>A*). (specific fragment of 1080 bp): 1) *CACA/TACA*; 2) *CGCG/TACA*; 3) *CAAG/CACG*; and 4) *CAAG/CACG*. **C.** PCR-SSP for rs3737002 (*1:g.207587428C>T*), rs11118131 (*1:g.207587851C>T*), (specific fragment of 457 bp); rs11118167 (*1:g.207608809T>C*), rs17047660 (*1:g.207609511A>G*): 1) (specific fragment of 746 bp): 1) *TCCA/TCTA*; 2) *CTCA/TCTA*; and 3) *CCCA/CTTA*. H: negative controls (complete reactions without DNA); bp: base pairs; HGH: Human Growth Hormone (as internal PCR control fragment of 431 bp for A and B); *HLA*-E: Human Leukocyte Antigen – E (as internal PCR control fragment of 324 bp).
