# Supplementary material for: Complement receptor 1 (CR1, CD35) association with susceptibility to leprosy
Source: PLoS Negl Trop Dis. 2018 Aug 9;12(8):e0006705. doi: 10.1371/journal.pntd.0006705 (PMC6103516; doi:10.1371/journal.pntd.0006705)
Supplement: S2 Fig — Colors are indicative of D'/logarithm of odds (LOD), and values correspond to r2. Bright red color represents LOD score for LD ≥ 2 and D = 1, shades of pink/red represents LOD ≥ 2 and D < 1, blue color represents D = 1 but LOD < 2, and white squares represent LOD< 2 and D < 1. A) Euro-Brazilians controls; B) Euro-Brazilians patients; C) Afro-Brazilian controls; D) Afro-Brazilian patients. Plots were constructed using Haploview 4.2. (PDF) [file pntd.0006705.s002.pdf]

ELETRONIC SUPPLEMENTARY MATERIAL

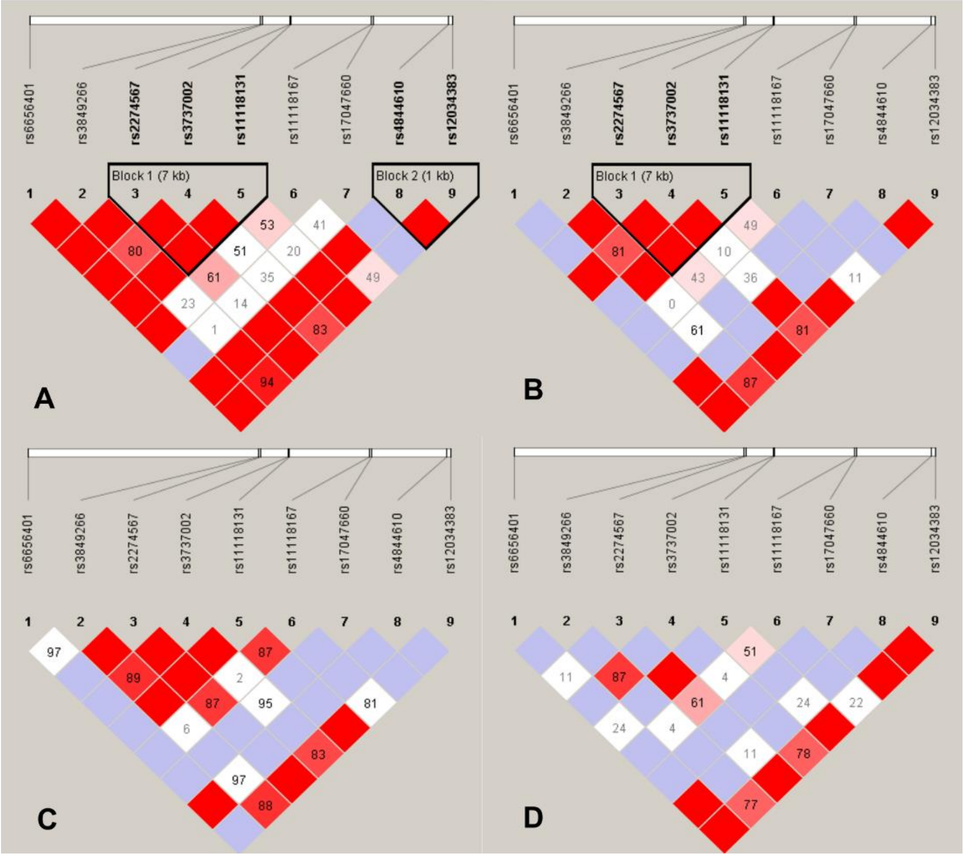

S2 Figure. Linkage disequilibrium between the investigated CR1polymorphisms.

Colors are indicative of D'/logarithm of odds (LOD), and values correspond to r². Bright red color represents LOD score for LD ≥ 2 and D = 1, shades of pink/red represents LOD ≥ 2 and D < 1, blue color represents D = 1 but LOD < 2, and white squares represent LOD< 2 and D < 1. A) Euro-Brazilians controls; B) Euro-Brazilians patients; C) Afro-Brazilian controls; D) Afro-Brazilian patients. Plots were constructed using Haploview 4.2.
