# Supplementary material for: Complement receptor 1 (CR1, CD35) association with susceptibility to leprosy
Source: PLoS Negl Trop Dis. 2018 Aug 9;12(8):e0006705. doi: 10.1371/journal.pntd.0006705 (PMC6103516; doi:10.1371/journal.pntd.0006705)
Supplement: S1 Table — Pat.: Patient; Con.: control; MB: multibacillary; PB: paucibacillary; N: samples; min.: minimum; med.:median; max.: maximum; P: P value for non parametric Mann-Whitney test; G + and AA correspond to SNP rs12034383. In bold: significant. (PDF) [file pntd.0006705.s003.pdf]

S1 Table. *CR1* gene expression in leprosy.

	**PAT**	**CON**	**p**	**MB**	**PB**	**p**	***G*⁺**	***AA***	**p**
**N**	45	31		7	37		33	9	
**Median**	2.10	1.63	0.813	1.41	2.20	0.245	2.39	1.25	**0.043**
**Min-Max**	0.39-7.28	0.31-6.19		0.51-3.58	0.39-7.28		0.39-7.28	0.53-2.80	

Pat.: Patient; Con.: control; MB: multibacillary; PB: paucibacillary; N: samples; min.: minimum; med.:median; max.: maximum; P: P value for non parametric Mann-Whitney test; *G* + and *AA* correspond to SNP rs12034383. In bold: significant.
