# Supplementary material for: Complement receptor 1 (CR1, CD35) association with susceptibility to leprosy
Source: PLoS Negl Trop Dis. 2018 Aug 9;12(8):e0006705. doi: 10.1371/journal.pntd.0006705 (PMC6103516; doi:10.1371/journal.pntd.0006705)
Supplement: S2 Table — Pat.: Patient; Con.: control; MB: multibacillary; PB: paucibacillary; N: samples; min.: minimum; med.:median; max.: maximum; P: P value for non parametric Mann-Whitney test; In bold: significant. (PDF) [file pntd.0006705.s004.pdf]

ELETRONIC SUPPLEMENTARY MATERIAL

S2 Table. Concentration of soluble CR1 in leprosy.

							rs12034383			rs3849266			rs3737002		
	**PAT**	**CON**	**p**	**MB**	**PB**	**p**	**A^+**	**GG**	**p**	**T^+(PB)**	**CC(PB)**	**p**	**T^+(PB)**	**CC(PB)**	**p**
**N**	58	22		30	28		21	13		13	15		16	12	
**Median.**	0.47	0.45	0.515	0.47	0.50	0.324	0.58	0.42	**0.023**	0.45	0.82	**0.040**	0.45	0.87	**0.046**
**Min-Max.**	0.07-3.82	0.09-0.98		0.07-3.82	0.08-2.85		0.33-3.82	0.07-0.92		0.08-0.96	0.09-2.85		0.08-0.98	0.09-2.85	

Pat.: Patient; Con.: control; MB: multibacillary; PB: paucibacillary; N: samples; min.: minimum; med.:median; max.: maximum; P: P value for non parametric Mann-Whitney test; In bold: significant.
