# Supplementary material for: Complement receptor 1 (CR1, CD35) association with susceptibility to leprosy
Source: PLoS Negl Trop Dis. 2018 Aug 9;12(8):e0006705. doi: 10.1371/journal.pntd.0006705 (PMC6103516; doi:10.1371/journal.pntd.0006705)
Supplement: S1 Checklist — (PDF) [file pntd.0006705.s006.pdf]

STROBE Statement—Checklist of items that should be included in reports of ***case-control studies***

| | Item No | Recommendation |
|---|---|---|
| **Title and abstract** | 1 | (*a*) Indicate the study's design with a commonly used term in the title or the abstract<br>We stated the following in the abstract: "*We investigated CR1 polymorphisms, gene expression and soluble CR1 levels in a case-control study with Brazilian leprosy patients…"* |
| | | (*b*) Provide in the abstract an informative and balanced summary of what was done and what was found.<br>What was done: see above and "*Nine polymorphisms were haplotyped by multiplex PCR-SSP in 213 leprosy patients (47% multibacillary) and 297 controls. mRNA levels were measured by qPCR and sCR1 by ELISA, in up to 80 samples*".<br>What was found: "*Individuals with the most common recombinant haplotype harboring rs3849266*T in intron 21 and rs3737002*T in exon 26 (encoding p.1408Met of the York Yka+ antigen), presented twice higher susceptibility to leprosy (OR=2.43, p=0.017). Paucibacillary patients with these variants presented lower sCR1 levels, thus reducing the anti-inflammatory response (p=0.046). Furthermore, the most ancient haplotype increased susceptibility to the multibacillary clinical form (OR=3.04, p=0.01) and presented the intronic rs12034383*G allele, which was associated with higher gene expression (p=0.043), probably increasing internalization of the parasite. Furthermore, there was an inverse correlation between sCR1 and MBL levels (R=-0.52, p=0.007).*" |
| **Introduction** | | |
| Background/rationale | 2 | Explain the scientific background and rationale for the investigation being reported.<br>From lines 59-64, we explain the epidemiological situation of the disease, underlining the importance of the investigation in public health. Pathology and state of the art of known genetic susceptibility to the disease are given in the following sentences, from 65-75. The importance of the complement system in leprosy is given from lines 76-84. |
| Objectives | 3 | State specific objectives, including any prespecified hypotheses<br>The hypothesis that *CR1* polymorphisms may modulate susceptibility to the disease is based on previous observations that the CR1 receptor is a key receptor for mycobacterial entrance (lines 85-86). Since only one case-control study was done until now, we outlined the importance of CR1 in the following sentences (87-98), proposing to investigate *CR1* polymorphisms, mRNA expression levels and sCR1 serum levels in Brazilian leprosy patients. |
| **Methods** | | |
| Study design | 4 | Present key elements of study design early in the paper.<br>This is a transversal case-control study, as already stated in the abstract and in the ethics statement. Key elements are given in the "Subjects and sample" section. |
| Setting | 5 | Describe the setting, locations, and relevant dates, including periods of recruitment, exposure, follow-up, and data collection<br>This information is given from lines 106-110, but exposure to mycobacteria normally occurs early in infancy and was not evaluated. Since this study is not longitudinal, there was no follow-up. |
| Participants | 6 | (*a*) Give the eligibility criteria, and the sources and methods of case ascertainment and control selection. Give the rationale for the choice of cases and controls.<br>Eligibility criteria is outlined from lines 110-112 for patients, and in lines 119-120 for |

| | | |
|---|---|---|
| | | controls. Ascertainment is described from lines 111-113. Cases were collected in leprosy reference centres, and controls were healthy volunteers or blood donors from the same centres or nearby blood banks, that may share a greater proportion of patient's environmental factors, including exposure to the parasite (it is estimated that around 70% of exposed individuals to *M. leprae* do not develop leprosy). This rationale is given in lines 115-117. |
| | | (*b*) For matched studies, give matching criteria and the number of controls per case<br>This study was unmatched; differences in demographic factors between both groups were corrected through logistic regression. |
| Variables | 7 | Clearly define all outcomes, exposures, predictors, potential confounders, and effect modifiers. Give diagnostic criteria, if applicable<br>Diagnostic criteria is given from line 113-115. Potential confounders (geographic origin, sex, age, ethnic group) are listed in Table 1. |
| Data sources/ measurement | 8* | For each variable of interest, give sources of data and details of methods of assessment (measurement). Describe comparability of assessment methods if there is more than one group<br>Genotyping results for all samples were obtained with multiplex PCR-SSP, as described in the topic "CR1 genotyping", Table 2 and S1 Figure. mRNA measurements were done with a subset of Sinop samples, using TaqMan RT-PCR (described in the next section "mRNA Quantification"), and soluble CR1 levels were measured with ELISA in a group of samples from Curitiba and Sinop, selected in order to achieve a balanced distribution of ethnic groups and genotypes (in the topic "sCR1 Quantification"). |
| Bias | 9 | Describe any efforts to address potential sources of bias<br>Differences in data distribution resulting from confounding factors, as geographic origin, age, sex and ethnicity, were statistically corrected using logistic regression. This is outlined in line 210-212. |
| Study size | 10 | Explain how the study size was arrived at<br>We calculated the sample size needed for detecting associations with allele frequencies of at least 10% with 95% confidence level and a confidence interval of 5.0 and arrived at minimal 384 chromosomes (at least 192 individuals). Our sample sizes achieved this minimum number, with the exception of the group of paucibacillary patients, which nevertheless was close to it (182). These explanatory sentences can be found at lines 208-212. |
| Quantitative variables | 11 | Explain how quantitative variables were handled in the analyses. If applicable, describe which groupings were chosen and why<br>Quantitative variables (*CR1* mRNA and soluble CR1 levels) were not normally distributed and thus compared using nonparametric methods (see below).<br>They were grouped into patients and controls, multi- and paucibacillary patients, to check if levels alter according to disease status and severity, and according to genotypes, to see if they influence gene and protein expression. Data were transformed into log10 for better graphical visualization. These information can be found in lines 221-226. |
| Statistical methods | 12 | (*a*) Describe all statistical methods, including those used to control for confounding<br>Statistical methods were described in lines 212-221, 226-228. Logistic regression was used for correcting association results for confounding factors as sex, age, geographic origin and ethnic group. |
| | | (*b*) Describe any methods used to examine subgroups and interactions |

| | | |
|---|---|---|
| | | The same methods outlined above were used for subgroups (multi and paucibacillary patients). |
| | | (*c*) Explain how missing data were addressed<br>We only included full-haplotyped samples in the logistic regression. |
| | | (*d*) If applicable, explain how matching of cases and controls was addressed<br>Not applicable. |
| | | (*e*) Describe any sensitivity analyses<br>There were none necessary for genotyping results. Inclusion of the outlier (excluded for better graphical visualization) did not change substantially the results of Mann-Whitney comparisons (they remained significant). |
| **Results** | | |
| Participants | 13* | (a) Report numbers of individuals at each stage of study—eg numbers potentially eligible, examined for eligibility, confirmed eligible, included in the study, completing follow-up, and analysed<br>Available numbers were given in the "Subjects and samples" section. |
| | | (b) Give reasons for non-participation at each stage<br>Not applicable. |
| | | (c) Consider use of a flow diagram<br>Not necessary for this study. |
| Descriptive data | 14* | (a) Give characteristics of study participants (eg demographic, clinical, social) and information on exposures and potential confounders<br>Given in Table 1. |
| | | (b) Indicate number of participants with missing data for each variable of interest<br>Subgroup numbers investigated for mRNA and sCR1 levels are included in supplementary tables 1 and 2. |
| Outcome data | 15* | Report numbers in each exposure category, or summary measures of exposure<br>Not applicable. |
| Main results | 16 | (*a*) Give unadjusted estimates and, if applicable, confounder-adjusted estimates and their precision (eg, 95% confidence interval). Make clear which confounders were adjusted for and why they were included<br>Results with these estimates are given in lines 258-266 and Table 3. |
| | | (*b*) Report category boundaries when continuous variables were categorized<br>Not applicable. |
| | | (*c*) If relevant, consider translating estimates of relative risk into absolute risk for a meaningful time period<br>Not applicable. |

| | | |
|---|---|---|
| Other analyses | 17 | Report other analyses done—eg analyses of subgroups and interactions, and sensitivity analyses<br>Outlined in lines 260-263, Tables S1 and S2. |
| **Discussion** | | |
| Key results | 18 | Summarise key results with reference to study objectives<br>Key results can be found in lines 236-327 |
| Limitations | 19 | Discuss limitations of the study, taking into account sources of potential bias or imprecision. Discuss both direction and magnitude of any potential bias<br>Limitations as sample sizes, genetic background, etc. were discussed in lines 302-304, 320-321, 327. |
| Interpretation | 20 | Give a cautious overall interpretation of results considering objectives, limitations, multiplicity of analyses, results from similar studies, and other relevant evidence<br>Interpretation of results are given in lines 236-327. |
| Generalisability | 21 | Discuss the generalisability (external validity) of the study results.<br>The final discussion can be found in lines 328-331. |
| **Other information** | | |
| Funding | 22 | Give the source of funding and the role of the funders for the present study and, if applicable, for the original study on which the present article is based<br>Funding information can be found in the funding statement. |

*Give information separately for cases and controls.

**Note:** An Explanation and Elaboration article discusses each checklist item and gives methodological background and published examples of transparent reporting. The STROBE checklist is best used in conjunction with this article (freely available on the Web sites of PLoS Medicine at http://www.plosmedicine.org/, Annals of Internal Medicine at http://www.annals.org/, and Epidemiology at http://www.epidem.com/). Information on the STROBE Initiative is available at http://www.strobe-statement.org.
